# Repeated Sigma-1 Receptor Antagonist MR309 Administration Modulates Central Neuropathic Pain Development After Spinal Cord Injury in Mice

**DOI:** 10.3389/fphar.2019.00222

**Published:** 2019-03-22

**Authors:** Sílvia Castany, Xavier Codony, Daniel Zamanillo, Manuel Merlos, Enrique Verdú, Pere Boadas-Vaello

**Affiliations:** ^1^ Research Group of Clinical Anatomy, Embryology and Neuroscience (NEOMA), Department of Medical Sciences, Universitat de Girona, Girona, Spain; ^2^ Esteve Pharmaceuticals, Drug Discovery and Preclinical Development, Parc Científic de Barcelona, Barcelona, Spain

**Keywords:** spinal cord injury, central neuropathic pain, MR309, central sensitization-related biomarkers, pro-inflammatory cytokines

## Abstract

Up to two-thirds of patients affected by spinal cord injury (SCI) develop central neuropathic pain (CNP), which has a high impact on their quality of life. Most of the patients are largely refractory to current treatments, and new pharmacological strategies are needed. Recently, it has been shown that the acute administration of the σ1R antagonist MR309 (previously developed as E-52862) at 28 days after spinal cord contusion results in a dose-dependent suppression of both mechanical allodynia and thermal hyperalgesia in wild-type CD-1 Swiss female mice. The present work was addressed to determine whether MR309 might exert preventive effects on CNP development by repeated administration during the first week after SCI in mice. To this end, the MR309 (16 or 32 mg/kg i.p.) modulation on both thermal hyperalgesia and mechanical allodynia development were evaluated weekly up to 28 days post-injury. In addition, changes in pro-inflammatory cytokine (TNF-α, IL-1β) expression and both the expression and activation (phosphorylation) of the N-methyl-D-aspartate receptor subunit 2B (NR2B-NMDA) and extracellular signal-regulated kinases (ERK1/2) were analyzed. The repeated treatment of SCI-mice with MR309 resulted in significant pain behavior attenuation beyond the end of the administration period, accompanied by reduced expression of central sensitization-related mechanistic correlates, including extracellular mediators (TNF-α and IL-1β), membrane receptors/channels (NR2B-NMDA) and intracellular signaling cascades (ERK/pERK). These findings suggest that repeated MR309 treatment after SCI may be a suitable pharmacologic strategy to modulate SCI-induced CNP development.

## Introduction

Spinal cord injury (SCI) global incidence is estimated as 40–80 new cases per million population per year (Officer et al., 2013). Approximately, a 60% of patients develop central neuropathic pain (CNP) which is a common complication after SCI (Siddall et al., 2003; Baastrup and Finnerup, 2012; Burke et al., 2017). Developing neuropathic pain has an impact on the quality of life, and it represents an economic burden associated with the primary care of the individual (Boakye et al., 2012; Shin et al., 2012). In order to deal with this health concern, several pharmacological treatments have been used targeting CNP alleviation (Vranken, 2013; Colloca et al., 2017; Widerström-Noga, 2017), but CNP is known to be largely refractory to these treatments (Dworkin et al., 2010; Finnerup et al., 2010; Burke et al., 2017). Therefore, it is necessary to address this unmet need by developing new pharmacological strategies toward coping central neuropathic pain.

In this regard, several preclinical studies have shown that sigma-1 receptor (σ1R) may play a critical role in the development of pathological pain (Merlos et al., 2017). Interestingly, the genetic ablation of σ1R in peripheral injury-induced pathological pain models resulted in either attenuation or avoidance of pain-related behaviors in mice (Cendán et al., 2005; de la Puente et al., 2009; Entrena et al., 2009a; Nieto et al., 2012; Tejada et al., 2014; Gris et al., 2015). In agreement with these results, it has recently reported that σ1R may play also a key role in the spinal cord injury-induced neuropathic pain development (Castany et al., 2018), since both thermal hyperalgesia and mechanical allodynia pain behaviors were markedly attenuated in σ1R KO mice subjected to spinal cord contusion. Moreover, this attenuation of spinal cord injury-induced neuropathic pain was associated with the lack of upregulation of central sensitization-related algogens (Castany et al., 2018), which was clearly evidenced in the spinal cord of injured wild-type mice. All these results provided grounds to consider the modulation of the σ1R as a suitable pharmacological approach to alleviate pathological pain associated with both peripheral and central pathologies involving central sensitization, including spinal cord injury.

Several structurally diverse compounds binding to the σ1R have been reported (Vela et al., 2015). σ1R ligands appear to be a suitable therapeutic approach as they are only active under pathological conditions, but inactive in resting conditions. For this reason, they have been studied for their therapeutic potential in neurodegenerative diseases or neuropathic pain conditions (Su and Hayashi, 2003; Tsai et al., 2009; Hayashi et al., 2011). Among the σ1R ligands, the σ1R antagonist MR309 (previously developed as E-52862) exerts antiallodynic and antihyperalgesic effects on several preclinical models involving sensitization of pain pathways (Nieto et al., 2012; Romero et al., 2012, 2016; Tejada et al., 2014; Gris et al., 2015, 2016). In fact, repeated treatment with MR-309 during the induction phase has been able to prevent the development of paclitaxel-induced neuropathy (Nieto et al., 2012). Focusing on SCI, the acute administration of MR309 at 28 days after contusion results in a dose-dependent suppression of both thermal hyperalgesia and mechanical allodynia in wild-type CD-1 Swiss female mice with similar ED50 values (Castany et al., 2018). Considering these promising results, it is not unreasonable to suggest that MR309 administration could be useful as a preemptive analgesic, which could modulate the establishment of a chronic pain state after SCI. Thus, the present study was addressed to determine whether MR309 might exert preventive effects on CNP development after SCI. To this end, two doses of MR309 were selected according to the ED50s obtained previously (Castany et al., 2018). Furthermore, in order to gain mechanistic insights, the spinal expression and phosphorylation of extracellular signal-regulated kinases (ERK1/2) and NMDA receptor NR2B subunit, as well as pro-inflammatory cytokines TNF-α and IL-1β (Ji et al., 2009; Wu and Zhuo, 2009; Ramesh, 2014), were investigated in parallel to the pain behavior evaluations, since all of them have been reported to be involved in CNP development.

## Materials and Methods

### Animals and Surgical Procedures

Five-week-old wild-type (WT) female CD1 mice that weighted a median of 22 g (19–26 g) were obtained from Charles River Laboratories (France). Mice were housed in a colony room at 21 ± 1°C and 40–60% humidity, with a 12:12 h light/dark cycle and access to food and water ad libitum, in groups of five in 331 × 159 × 132 mm plexiglass cages with a wood-shaving bedding. Cages were changed twice weekly. Behavioral testing was carried out in a soundproof experimental room. All mice were allowed to acclimatize to the facility rooms before commencing any behavioral or surgical procedures, which were all conducted during the light cycle. Sentinel mice were routinely tested for pathogens, and facilities remained pathogen-free during the whole experimental period.

All the experimental procedures and animal husbandry were conducted following the ARRIVE guidelines and according to the I.A.S.P. ethical principles for the evaluation of pain in conscious animals and the European Parliament and the Council Directive of 22 September 2010 (2010/63/EU) and were approved by the Animal Ethics Committee of the Parc Científic of Barcelona.

### Drugs

MR309 (4-[2-[[5-methyl-1-(2-naphthalenyl)-1H-pyrazol-3-yl]oxy]ethyl]morpholine) was used as hydrochloride, and doses were expressed as weights of this form. MR309 was synthesized by Laboratorios Esteve (Barcelona, Spain). The analgesic drug was suspended in an aqueous solution (0.5% hydroxypropylmethyl cellulose, HPMC; Sigma-Aldrich) and administered by the intraperitoneal (i.p.) route at a volume of 10 ml/kg.

### Experimental Design and Dosing

The present study was aimed to evaluate the preventive effects of MR309 administration on SCI-induced neuropathic pain development. The preventive pharmacological protocol consisted in the administration of two doses of MR309 (16 or 32 mg/kg) or vehicle (HPMC, hydroxypropyl methylcellulose) in independent sets of animals. Sham groups with HPMC or the high dose of MR309 (32 mg/kg) administration were included as controls. The administration for either MR309 doses or vehicle started 30 min immediately after the SCI. It was administered twice a day during the first week after spinal cord injury. Once the drug or vehicle administration ended at 7 days post-injury (dpi), locomotor (BMS) and nociceptive evaluation (mechanical allodynia and thermal hyperalgesia) were performed in this particular order at 7, 14, 21, 28 dpi to evaluate the effect of the early administration of the studied drug in these different outcomes. The selected time points were chosen based on both the ability of the mice to have suitable locomotor performance and painful phenotype established for pain evaluation (Nakae et al., 2011; Castany et al., 2018).

To study the underlying molecular changes exerted by MR309 in the spinal cord, different experimental groups were dissected at two different time points of the study (14 and 28 dpi). Protein expression of some pain biomarkers was evaluated, including pERK phosphorylation, NMDAR phosphorylation at S1303 and Y1472 sites, and the proinflammatory cytokines TNF-α and IL1β.

Functional and molecular measurements were performed in a blinded manner using a code for both mice and samples.

### Surgical Procedure

In order to obtain a mouse model of CNP without locomotor paralysis, spinal cord contusion was conducted according to procedures explained elsewhere (Kuhn and Wrathall, 1998; Álvarez-Pérez et al., 2016; Castany et al., 2018). Briefly, animals were anesthetized with sodium pentobarbital (50 mg/kg, i.p.) and placed prone on a heating pad to maintain constant body temperature. After back disinfection with povidone iodine, T8–T9 of the thoracic spinal cord was exposed *via* dorsal laminectomy, a metallic stage positioned over the exposed spinal cord and a 2 g weight then dropped onto the stage from a 25 mm height. Following this procedure, the wound was closed, and animals were allowed to recover in warmed cages with accessible food and water. After the surgical procedure, animals also received 0.5 ml saline solution (i.p.) to restore an eventual volemic deficit. In sham animals, the spinal cord was exposed as described above but not contusioned, and they underwent the same recovery procedures. Naïve mice did not receive any surgical manipulation. Mice were randomly allocated to experimental groups prior to surgical procedures.

### Locomotor Activity Evaluation

Locomotor activity was evaluated by means of the Basso Mouse Scale for locomotion (BMS) test and performed as described elsewhere (Álvarez-Pérez et al., 2016; Castany et al., 2018). Briefly, animals were allowed to freely move inside an open field (72 cm × 72 cm) during 5 min, and the mouse hind-limb movements were scored the locomotor function according to the Basso Mouse Scale (Basso et al., 2006), which ranges from 0 (no hind-limb movement) to 9 (normal movement-coordinated gait).

### Mechanical Allodynia and Thermal Hyperalgesia Tests

Mechanical allodynia was quantified by measuring the hind paw withdrawal response to von Frey filament stimulation (Chaplan et al., 1994; Castany et al., 2018). Mice were placed in compartment enclosures in a test chamber with a framed metal mesh floor through which von Frey monofilaments (bending force range from 0.04 to 2 g) were applied onto the plantar surface, and thresholds were measured using the up-down method paradigm. The filament of 0.4 g was used first. Then, the strength of the next filament was decreased when the animal responded or increased when the animal did not respond. This up-down procedure was stopped four measures after the first change in animal responding. The application each filament was applied 2 s at intervals of about 5–10 s between each stimulation. Clear paw withdrawal, shaking or licking was considered as a nociceptive-like response. Both hind paws were tested. The mechanical threshold that produced 50% of responses was calculated using the Dixon formula: 50% paw withdrawal threshold (g) = [(10(*Xf* + *κδ*)/10,000)], where *Xf* is the value (in logarithmic units) of the final von Frey filament used, *k* is a fixed tabular value for the pattern of positive/negative responses, and *d* is the mean difference (in log units) between stimuli.

Thermal hyperalgesia was assessed using the plantar test analgesia meter (IITC, Life Science) by determination of hind paw withdrawal latency in response to a thermal stimulus (radiant heat). The plantar test was performed according to the Hargreaves method (Hargreaves et al., 1988; Castany et al., 2018). Mice were placed into compartment enclosure on the tempered (29°C) glass surface of the plantar test device and allowed to acclimate for 45 min. Radiant heat source was positioned under the plantar surface of the hind paw and activated with a light beam intensity chosen in preliminary studies to give baseline latencies of 14–15. A cut-off time of 20 s was imposed in order to prevent damage in the absence of response. The mean withdrawal latencies for both hind paws were determined from the average of three separated trials, taken at 5 min interval. Both paws were evaluated since SCI model results in a bilateral injury and it is not possible to use contralateral paw as a natural intraindividual control.

### Western Blotting

Immediately after anesthesia with sodium pentobarbital (100 mg/kg,i.p.), spinal cord segments of the lesion site (T8–T9) were carefully removed from WT mice (*n* = 4–6 per group) at 14 and 28 days after surgery and frozen immediately in dry ice and stored at −80°C. Spinal cord tissue was homogenized by sonication in TRIS buffer (50 mM Tris, 150 nM NaCl, 1% NP-40, 2 mM EDTA, 1 mM phenylmethylsulfonyl fluoride, Triton X-100, 0.1% SDS, 1 mM Na_3_VO_4_, 25mM Na), 5% protease inhibitor cocktail, 1% phosphatase inhibitor cocktail, all from Sigma-Aldrich Química S.A. The resultant homogenate was then centrifuged at 10,000*g* at 4°C for 10 min. The supernatant was decanted from the pellet, and the protein concentration from the obtained supernatant was measured using a Lowry assay. Samples were then stored at −80°C until use. Thirty micrograms were fractionated by 10% (w/v) SDS-PAGE and transferred onto a polyvinylidene difluoride membrane, then blocked, with either 5% nonfat dry milk or bovine serum albumin (BSA), in Tris-Tween 20-buffered saline (T-TBS) for 1 h at room temperature. Membranes were then incubated with primary antibodies overnight at 4°C: rabbit anti extracellular signal-regulated kinases (total ERK 1/2) (1:40,000, M5670, Sigma-Aldrich) and diphosphorylated ERKs (pERK1/2) (1:800, 44680G, invitrogen) were diluted in T-TBS containing 1% nonfat dry milk. Rabbit anti-pY1472-GluN2B (1:1,000, M2442, Sigma-Aldrich), anti-pS1303-GluN2B (1:3,000, ab81271, Abcam), anti-GluN2B (1:750, ab15557P, Merck Millipore), anti-TNF-α (1/500, ab6671, Abcam), and anti-IL-1β (1/500, ab9722, Abcam) were also used and diluted in T-TBS containing 1% BSA solution. To ensure equal protein loading, rabbit anti-GAPDH antibody (1:40,000, Sigma-Aldrich Química S.A.) was used as a loading control. The blots were washed four times for 15 min with T-TBS and then incubated for 1 h at room temperature with horseradish peroxidase-conjugated goat antirabbit IgG, purchased from Pierce Biotechnology Inc. (Rockford, IL, USA) and revealed by chemiluminescence (Immun-Star HRP Chemiluminescent Kit) from Bio-Rad. Chemiluminescence was detected with the ChemiDoc XRS System from Bio-Rad. The densiometric analysis of immunoreactive bands was done using the Image Studio Lite 5.2 (LI-COR Bioscience). pERK, Y1472-GluN2B, and pS1302-GluN2B were normalized to total ERK and total gluN2B, respectively, and in turn, normalized with respect to the intensity of the corresponding GAPDH immunoreactivity. TNFα and IL1β were also normalized to the corresponding GAPDH intensity. Original images of Western blot are supplied in Supplementary Material Figures.

### Statistical Analysis

Functional and biochemical measurements were performed in a blinded manner using a code for both mice and samples. All data are expressed as the mean ± SEM. Data were analyzed using repeated measures MANOVA (Wilks’ criterion) and analysis of variance (ANOVA) followed by Duncan’s test, when applicable. Molecular results obtained by western blotting are expressed as the percentage of change when compared to the control group (Sham animals treated with HPMC).

## Results

### General Observations

Following a protocol animal welfare supervision based on Morton and Griffiths (1985) guidelines, changes in coat and skin, vibrissae of nose, nasal secretions, signs of autotomy of hindpaw and/or forepaw, or aggressiveness were not detected in mice after SCI at any time of the experimental period. Moreover, the animals showed no significant weight loss throughout the experiment.

### MR309 Treatment Attenuates Both Mild Spinal Cord Injury-Induced Mechanical Allodynia and Thermal Hyperalgesia Development

**Figure 1:**
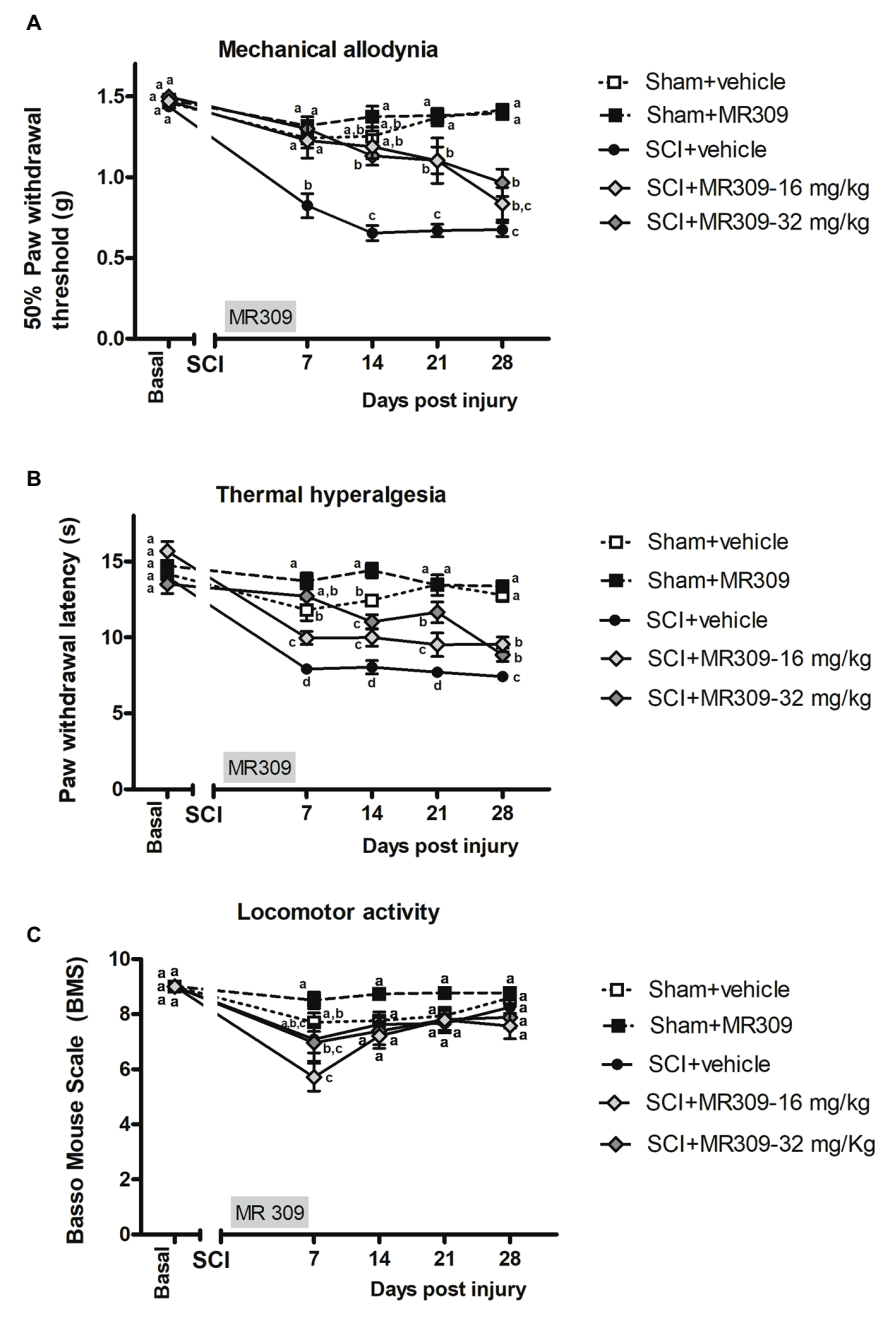
Time-course assessment of mechanical allodynia, thermal hyperalgesia, and locomotor activity after preventive sigma-1 receptor antagonist MR309 treatment. Each point and vertical line represent the mean ± SEM. Experimental groups: Sham-Veh (*n* = 17), Sham-MR309 (*n* = 13), SCI-Veh (*n* = 12), SCI-MR309-16 mg/kg (*n* = 7), and SCI-MR309-32 mg/kg (*n* = 13). a–c: groups not sharing a letter are significantly different, *p* < 0.05, by Duncan’s test **(A)** The MANOVA analysis of mechanical allodynia indicated significant effects on day (*F*_(4,54)_ = 66.08, *p* < 0.001), treatment (*F*_(4,57)_ = 46.59, *p* < 0.001), and interaction for day × treatment factors (*F*_(16,165)_ = 8.53, *p* < 0.001). Significant group differences were found on post-injury days 7, 14, 21, and 28 (all *p*’s < 0.001) by ANOVA analysis. On the whole, mechanical allodynia is prevented in treated SCI-animals up to 14 dpi and attenuated until the end of experimental period. **(B)** The MANOVA analysis of thermal hyperalgesia indicated significant effects on day (*F*_(4,58)_ = 55.59, *p* < 0.001), treatment (*F*_(4,61)_ = 47.01, *p* < 0.001), and interaction for day × treatment factors (*F*_(16,177)_ = 7.66, *p* < 0.001). Significant group differences were found on post-injury days 7, 14, 21, and 28 (all *p*’s < 0.001) by ANOVA analysis. Note that thermal hyperalgesia is prevented in MR309-32 mg/kg animals up to 7 dpi. Both doses significantly attenuate thermal hyperalgesia development up to 28 dpi. **(C)** The MANOVA analysis of BMS indicated significant effects on day (*F*_(4,54)_ = 23.57, *p* < 0.001), treatment (*F*_(4,57)_ = 5.01, *p* = 0.002) factors, and a lack of significant interaction for day × treatment (*F*_(16,168)_ = 1.63, *p* = 0.089). Significant group differences were identified only at 7 dpi by ANOVA analysis. Considering BMS scale parameters, mild BMS alterations referring to altered paw position but not to altered horizontal locomotion, at 7 dpi. No significant differences between groups from 14 to 28 dpi.

The repeated measure analysis of mechanical allodynia along the experimental period indicated significant effects on day (*p* < 0.001) and treatment (*p* < 0.001) factors and significant interaction for day × treatment (*p* < 0.001). Furthermore, significant group differences were found on post-injury days 7, 14, 21, and 28 (all *p*’s < 0.001) on further ANOVA analysis. Concretely, at 7 days post-injury (dpi), spinal cord injured mice without treatment (SCI-Veh) showed significant decrease in paw withdrawal mechanical thresholds when compared with all other experimental groups (*p* < 0.05) (Figure 1A). Similarly, at 14 dpi animals treated with 16 mg/kg of MR309 (SCI-MR309-16) or 32 mg/kg of MR309 (SCI-MR309-32) showed a significant increase of paw withdrawal mechanical thresholds when compared with SC-Veh (*p* < 0.05). Moreover, although injured groups treated with MR309 showed slight significant differences in comparison to sham treated with 32 mg/kg of MR309 (Sham-MR309), no significant differences were detected between Sham-Veh and SCI-MR309-16 or SCI-MR309-32. Then, at 21 and 28 dpi, both treated groups SCI-MR309-16 and SCI-MR309-32 showed significant mechanical allodynia attenuation when compared to SCI-Veh (*p* < 0.05). Finally, injured mice treated with vehicle showed increased pain responses in comparison to both sham groups. Hence, the mechanical allodynia developed in SCI-Veh mice was clearly prevented trough SCI-MR309-16 and SCI-MR309-32 treatments up to 14 dpi, and both treatments exerted an attenuation of mechanical allodynia development at 21 dpi becoming a mild attenuation at the end of the experimental period.

Similar to mechanical allodynia, the repeated measure analysis indicated significant effects on day (*p* < 0.001) and treatment (*p* < 0.001) factors and significant interaction for day × treatment (*p* < 0.001). Moreover, significant group differences were found on post-injury days 7, 14, 21, and 28 (all *p*’s < 0.001) on further ANOVA analysis. Concretely, at 7 dpi, SCI-MR309-32 mice in addition to showing reduced hyperalgesia in comparison with SCI-Veh, did not show significant differences with either Sham-Veh or Sham-MR309 groups (Figure 1B), indicating preventing effects on thermal hyperalgesia development. Moreover, although SCI-MR309-16 also showed reduced hyperalgesia when compared with SCI-Veh, this reduction was significantly lower than the exerted by the treatment of 32 mg/kg MR309. From 14 to 28 dpi, spinal cord injured mice treated with vehicle showed a significant increase hyperalgesia in comparison to both sham groups (*p* < 0.05). Moreover, both SCI-MR309-16 and SCI-MR309-32 groups showed significant thermal hyperalgesia attenuation when compared to SCI-Veh (*p* < 0.05). It is worth noting that SCI-MR309-32 also showed a significant increase in paw withdrawal latency to thermal stimulation when compared to SCI-MR309-16 at 21 dpi (*p* < 0.05), but this difference was not observed at 28 dpi, when both treated groups showed similar thermal hyperalgesia attenuation. Thus, the thermal hyperalgesia developed in SCI-Veh mice was clearly prevented through SCI-MR309-32 treatment up to 7 dpi. Moreover, both doses SCI-MR309-32/16 treatment attenuated thermal hyperalgesia development up to 21 dpi and produced a mild but significant attenuation by 28dpi.

In parallel, locomotor analysis by BMS test suggested no major impairment in coordination and locomotor function associated with SCI since all animals showed above six rating scores at 7 dpi and above 7 from 14 to 28 dpi (Figure 1C). The repeated measure analysis of BMS indicated significant effects on day (*F*_(4,54)_ = 23.57, *p* < 0.001) and treatment (*F*_(4,57)_ = 5.01, *p* = 0.002) factors and a lack of significant interaction for day × treatment (*F*_(16,168)_ = 1.63, *p* = 0.089). However, significant group differences were identified by ANOVA only on post-injury day 7, where SCI-treated mice showed a significant decreased BMS scores when compared with Sham-MR309. At the following days from 14 to 28 dpi, ANOVA analysis revealed no differences between experimental groups, stating that the mild spinal cord contusion did not result in paralysis or severe immobility. Hence, all mice groups were able to move freely without major locomotor interferences on functional evaluations.

Overall, functional data clearly show that preventive treatment with MR309 during the first week post-injury significantly attenuates mechanical and thermal hypersensitivity induced by a mild spinal cord contusion. In addition, changes in coat and skin, vibrissae, nasal secretions, autotomy signs of hind-paw and/or forepaw, or aggressiveness were not detected either in any experimental group or at any time of the study period.

### MR309 Treatment Modulates Central Sensitization-Related Molecular Biomarkers in Spinal Cord Injured Mice

**Figure 2:**
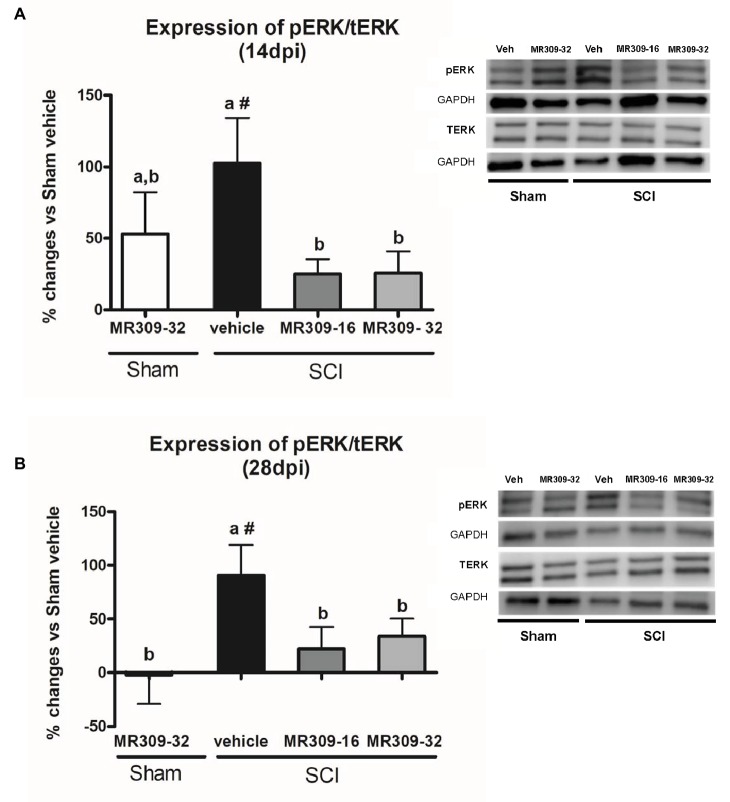
Spinal ERK1/2 phosphorylation (pERK) expression after preventive sigma-1 receptor antagonist MR309 treatment. Quantification and representative immunoblots of total ERK (tERK), pERK, and glyceraldehyde 3-phosphate dehydrogenase (GAPDH). Experimental groups: Sham-Veh (*n* = 5), Sham-MR309-32 (*n* = 5), SCI-Veh (*n* = 4), SCI-MR309-16 (*n* = 5), and SCI-MR309-32 (*n* = 5). Protein expressions were normalized to GAPDH, and data are presented as a percentage respect to SCI-Veh mice. ANOVA analysis revealed significant differences at both 14 (*F*_(4,23)_ = 3.54, *p* = 0.025) and 28 (*F*_(4,24)_ = 3.65, *p* = 0.022) days post-injury. a, b: groups not sharing a letter are significantly different, *p* < 0.05, by Duncan’s test; #: significant differences vs. SCI-Veh (*p* < 0.05, Duncan’s test). MR309 treatments prevent pERK upregulation observed in mild spinal cord injured mice at both 14 **(A)** and 28 **(B)** days post-injury. Control images were reused either for illustrative purposes or methodological purposes when several protein levels were assessed in one blot. Full-length blots are presented in Supplementary Figure S1.

Significant group differences were detected in ERK1/2 phosphorylation (pERK1/2) at both 14 (*p* = 0.025) and 28 (*p* = 0.022) days post-injury. A transient increase in pERK1/2 was detected in Sham-MR309 group at 14 dpi. However, treatment with both doses of MR309 in injured mice prevented the pERK1/2 upregulation detected in SCI-Veh either at 14 (Figure 2A) or 28 dpi (Figure 2B), showing no significant differences with Sham-Veh groups. These results indicate that activation/phosphorylation of ERK was significantly prevented after MR309 preventive treatment. Moreover, no significant changes (*p* > 0.05) in total ERK protein in spinal cord injured mice was detected when compared with sham-operated groups.

**Figure 3:**
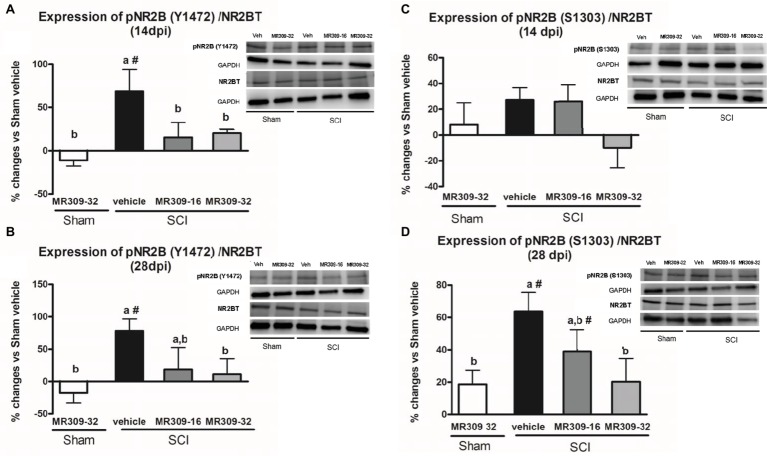
Spinal Tyr1472 and Ser1303 phosphorylation of N-methyl-D-aspartate (NMDA) receptor NR2B subunit after preventive sigma-1 receptor antagonist MR309 treatment. ANOVA analyses revealed significant group differences in pNR2B-Tyr1472 at both 14 (*F*_(4,23)_ = 4.39, *p* = 0.011) and 28 (*F*_(4,24)_ = 3.52, *p* = 0.025) dpi, and in pNR2B-Ser1303 at 28 dpi (*F*_(4,19)_ = 4.83, *p* = 0.01), but not at 14 dpi (*F*_(4,19)_ = 1.68, *p* = 0.206). **(A,B)** Quantification and representative immunoblots of total NR2B, pY1472NR2B, and glyceraldehyde 3-phosphate dehydrogenase (GAPDH) at 14 and 28 days post-injury. **(C,D)** Quantification and representative immunoblots of total NR2B, pS1303NR2B, and GAPDH at 14 and 28 days post-injury. Experimental groups: Sham-Veh (*n* = 5), Sham-MR309 (*n* = 5), SCI-Veh (*n* = 5), SCI-MR309-16 (*n* = 5), and SCI-MR309-32 (*n* = 5). Protein expressions were normalized to GAPDH, and data are presented as a percentage respect to SCI-Veh mice. a, b: groups not sharing a letter are significantly different, *p* < 0.05, by Duncan’s test; #: significant differences vs. SCI-Veh (*p* < 0.05, Duncan’s test). pY1472NR2B upregulation after SCI was prevented by both doses of MR309 (16 and 32 mg/kg), whereas pS1303NR2B was decreased by both doses of MR309 (16 and 32 mg/kg) at 28 days post-injury. Control images were reused either for illustrative purposes or methodological purposes when several protein levels were assessed in one blot. Full-length blots are presented in Supplementary Figure S2.

On the other hand, regarding NMDA receptor phosphorylation, significant group differences were detected in pNR2B-Tyr1472 at both 14 (*p* = 0.011) and 28 (*p* = 0.025) dpi. On both days, only SCI-Veh group showed significant increase in phosphorylation at Tyr1472 in NR2B subunit compared to other groups (Figures 3A,B), indicating that this phosphorylation was significantly prevented by means of MR309 treatment. As for pNR2B-Ser1303, group differences were shown at 28 dpi (*p* = 0.01), but not at 14 dpi (*p* = 0.206) (Figures 3C,D). Specifically, at 28 dpi, SCI-MR309-32 group shows a completely prevention of the pNR2B-Ser1303 upregulation when compared to SCI-Veh, whereas SCI-MR309-16 group showed a mild attenuation of NR2B-Ser1303 phosphorylation. No significant changes in the total levels of NMDAR-NR2B were observed in any of the experimental groups.

Overall, these findings suggest that MR309 treatment applied during the first week post-injury modulates the expression of pERK1/2 and pNR2B-NMDA, which are significantly upregulated after SCI in untreated mice.

### MR309 Treatment Prevents Pro-Inflammatory Cytokines TNF-α and IL-1β Upregulation in Spinal Cord Injured Mice

**Figure 4:**
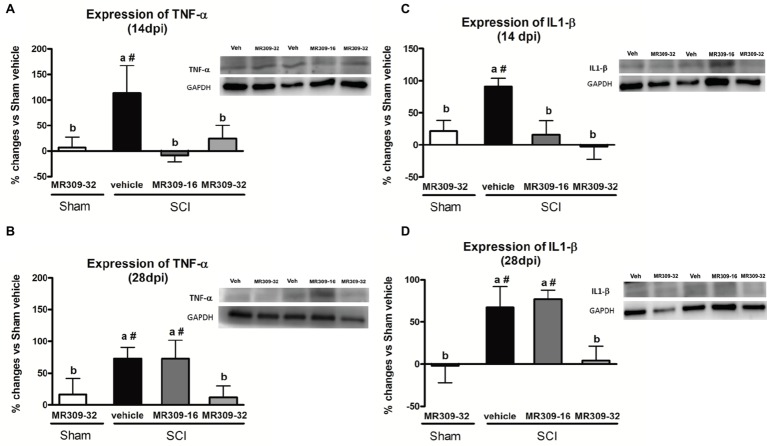
Spinal inflammatory cytokines [tumor necrosis factor-α (TNF-α) and interleukin-1β (IL-1β)] expression after preventive sigma-1 receptor antagonist MR309 treatment. The ANOVA analysis revealed significant differences between groups in TNF-α and IL-1β expression at 14 (TNF-α *F*_(4,23)_ = 2.91, *p* = 0.049; IL-1β *F*_(4,22)_ = 5.25, *p* = 0.006) and 28 (TNF-α *F*_(4,18)_ = 3.43, *p* = 0.037; IL-1β *F*_(4,24)_ = 6.26, *p* = 0.002) dpi **(A,B)** Quantification and representative immunoblots of TNF-α and glyceraldehyde 3-phosphate dehydrogenase (GAPDH) at 14 and 28 days post-injury. **(C,D)** Quantification and representative immunoblots of IL-1β and GAPDH at 14 and 28 days post-injury. Experimental groups: Sham-Veh (*n* = 5), Sham-MR309 (*n* = 5), SCI-Veh (*n* = 5), SCI-MR309-16 (*n* = 5), and SCI-MR309-32 (*n* = 5). Protein expressions were normalized to GAPDH, and data are presented as a percentage respect to SCI-Veh mice. a, b: groups not sharing a letter are significantly different, *p* < 0.05, by Duncan’s test; #: significant differences vs. SCI-Veh (*p* < 0.05, Duncan’s test). Both MR309 doses prevented TNF-α and IL-1β upregulation at 14 days post-injury. However, MR309 dose of 32 mg/kg, but not MR309 dose of 16 mg/kg, exerted this effect at 28 days post-injury. Control images were reused either for illustrative purposes or methodological purposes when several protein levels were assessed in one blot. Full-length blots are presented in Supplementary Figure S3.

Significant differences were shown between groups in TNF-α expression at 14 (*p* = 0.049) and 28 (*p* = 0.037) dpi (Figures 4A,B). At 14 dpi, MR309 treatments clearly prevent TNF-α upregulation since both SCI-MR309-32 and SCI-MR309-16 groups showed a significant decrease expression when compared with SCI-Veh group. At 28 days, only the MR309-32 group showed a significant decrease in the overexpression of TNF-α when compared to the SCI-vehicle. These results suggest that preventive treatment of 32 mg/kg of MR309 may prevent TNF-α overexpression observed in spinal cord injured mice up to 28 dpi, and 16 mg/kg of MR309 exert this effect up to 14 dpi.

Similarly, significant differences were detected between groups in IL-1β expression at 14 (*F*_(4,22)_ = 5.25, *p* = 0.006) and 28 (*F*_(4,24)_ = 6.26, *p* = 0.002) dpi (Figures 4C,D). At 14 dpi, MR309 treatments clearly prevent IL-1β upregulation since both SCI-MR309-32 and SCI-MR309-16 groups showed significant decrease in IL1-β overexpression when compared with SCI-Veh group. At 28 dpi, SCI-MR309-32 group still show significantly decreased IL-1β expression in comparison SCI-Veh, but not for injured mice treated with SCI-MR309-16 treatment. These results indicate that the treatment of 32 mg/kg of MR309 prevents IL-1β upregulation associated with SCI up to 28 dpi, and 16 mg/kg of MR309 exert this effect up to 14 dpi.

In summary, all these findings suggest that MR309 treatment applied during the first week post-injury may modulate the expression of central sensitization-related pro-inflammatory cytokines, which are upregulated in untreated spinal cord injured mice.

## Discussion

The present study was aimed to evaluate whether preventive administration of MR309 may be a suitable pharmacological strategy to modulate SCI-induced CNP development. Our findings indicated that the administration of both doses (16 or 32 mg/kg) of MR309 during the first week after SCI prevent mechanical allodynia development until 14 dpi, followed by an attenuation from 21 to 28 dpi. Thermal hyperalgesia was also prevented at 7 dpi and attenuated from 14 to 28 dpi.

The observed antinociceptive effects of MR309 are consistent with previous studies performed on either peripheral neuropathic pain or inflammatory pain models using repeated treatment of σ1R antagonists. Some of them were aimed to evaluate the effect of σ1R antagonists once the neuropathic pain is already established (Gris et al., 2016; Paniagua et al., 2017). However, other studies similar to our experimental design, consisting of repeated administration of σ1R antagonist starting the same day of injury induction is also reported.

Romero et al. (Romero et al., 2012) demonstrated that the administration of MR309 during 20 days after partial sciatic nerve ligation (PSNL) surgery in mice resulted in a reduction of nociceptive behaviors. However, when the treatment was suspended, nociceptive thresholds returned to those of vehicle-treated animals suggesting that in this particular model, a continuous σ1R antagonism is needed to maintain efficacy (Romero et al., 2012). On the contrary and similar to our results, the administration of the σ1R antagonist BD-1047 on postoperative days 0–5 in a preventive protocol reduced significantly mechanical allodynia in rats subjected to chronic constriction injury (CCI) and chronic compression of dorsal root ganglia (CCD) not only during the treatment period but also throughout the experimental period (30 and 14 dpi, respectively) (Roh et al., 2008; Son and Kwon, 2010). Particularly relevant is the case of paclitaxel-induced neuropathy, in which the co-administration of paclitaxel and the σ1R antagonists BD-1063 or MR-309 during 5 days totally prevented the development of paclitaxel-induced cold allodynia, at least until 3 weeks after first paclitaxel administration (Nieto et al., 2012). Therefore, our findings are in accordance with those observed in the CCI, CCD, and paclitaxel models, since the attenuation of SCI-induced CNP by MR-309 was observed until the last experimental day, far beyond the last drug administration. Furthermore, the present study is the first to show that repeated treatment of MR309 in SCI mice attenuates both thermal hyperalgesia and mechanical allodynia development up to 28 days post-injury, corresponding to the acute phase injury period. It has recently been reported that patients with oxaliplatin chemotherapy-induced peripheral neuropathy treated with repeated MR309 (daily for 5 days in each cycle of chemotherapy up to a maximum of 12 cycles) showed a significant reduction of cold pain threshold temperature and suprathreshold cold stimulus-evoked pain intensity. In addition, the proportion of patients with severe chronic neuropathy was significantly lower in the MR309 group (Bruna et al., 2018). These findings and our results suggest that repeated administration of MR309 is an appropriate treatment regimen to treat and prevent the development of both peripheral and central neuropathic pain.

Overall, our results suggest a long-lasting effect of MR309 treatment in SCI-induced CNP and further support the importance of an early blockade of the σ1R in preventing neuropathic pain development. However, since the attenuation efficacy of MR309 seems to slightly decrease at the end of the experimental period, it is worth to mention that new administration protocols may be studied. For instance, a future approach would be to assess the effects of MR309 in a longer protocol administration during the induction phase of the disease. On the other hand, it is worth to note that SCI-induced neuropathic pain has been predominantly studied in young male rodents (Kramer et al., 2017; Shiao and Lee-Kubli, 2018). The present work was focused in SCI-female model since epidemiological data highlight a higher prevalence of chronic pain in female which also show a higher vulnerability in the development of comorbid pain and emotional disorders (Miller and Cano, 2009; Goesling et al., 2013). Considering that CNP emerges within the first year after SCI, and it tends to become chronic because of the lack of efficient treatments (Siddall et al., 2003; Ataoğlu et al., 2013; Finnerup et al., 2014), it was necessary to evaluate not only a new therapeutic strategy addressed to attenuate CNP development during the acute phase of SCI but also an efficient one in females, which are more vulnerable to develop health issues associated with pathological pain.

In order to elucidate the MR309 effects on molecular pathways leading to attenuation of CNP development, we focused on key central sensitization-related biomarkers in the spinal cord. Regarding intracellular signaling cascades, we analyzed the expression and activation/phosphorylation of ERK1/2, which is known to be involved in central/spinal sensitization, especially after nervous system traumatic injuries (Yu and Yezierski, 2005; Crown et al., 2006; Cruz et al., 2006; Castany et al., 2018). Indeed, Castany et al., 2018 recently revealed that the CNP attenuation observed in the σ1R knockout mice subjected to spinal cord contusion was associated with pERK downregulation suggesting that the presence of σ1R facilitates ERK activation in the injured spinal cord. Consistent with these results, the upregulation of pERK detected at both 14 and 28 dpi after SCI is completely prevented by both doses of MR309 (16 and 32 mg/kg). This may indicate that early treatment with MR309 for 7 days is effective enough, at 14 and 28 dpi, to avoid the pathological increase in intracellular Ca^2+^ that will lead to activation of several MAPK and the final enhancement of the ERK pathway signaling. In addition, Castany et al., 2018 also showed that pain-related behaviors in σ1R knockout SCI-mice occurred concomitantly with a substantial reduction in the spinal cord NMDA phosphorylation, particularly at subunit NR2B, Tyr1472, and Ser1303, which are mainly related to NMDA receptor stabilization (Abe et al., 2005; Chen and Roche, 2007) and potentiation (Mao et al., 2014), respectively. These results suggest that σ1R facilitates NMDA receptor sensitization in the spinal cord in the SCI model of neuropathic pain, and that inhibition of NMDA receptor phosphorylation in mice lacking σ1R may contribute to the attenuated hypersensitivity to mechanical and thermal stimuli after SCI. In the present study, the phosphorylation at Y1472 was increased in animals subjected to SCI at 14 and 28 dpi, but, while both doses of MR309 were able to prevent phosphorylation at 14 dpi, only the high dose (32 mg/kg) was able to prevent Y1472 NR2B-phosphorylation at the end of the experiment period (28 dpi). Of note, the low dose of 16 mg/kg was only effective to modulate short-term changes. In regard of phosphorylation at S1303 site, preventive treatment with both doses failed to induce any changes at 14 dpi, as no elevated levels of phosphorylation in injured mice treated with vehicle were detected. However, at 28 dpi only the high dose (32 mg/kg) used was able to reduce the increased phosphorylation observed in SCI vehicle mice. Altogether, these results allow suggesting that MR309, through preventing upregulation of phosphoS1303 and phosphoY1472 NR2B, will decrease the stability of active NMDAR and their permeability further avoiding spinal synaptic plasticity and sensitization and contributing to the attenuated hypersensitivity detected in SCI-treated mice.

Pro-inflammatory cytokines TNF-α and IL-1β play a major role in pain and central sensitization phenomena in the context of CNP (Ji and Suter, 2007; Gustafson-Vickers et al., 2008; Zhang et al., 2011; Clark et al., 2013; Morioka et al., 2015; Álvarez-Pérez et al., 2016; Anwar et al., 2016). TNFα induces mechanical hypersensitivity and increased glutamatergic neurotransmission (Morioka et al., 2015), as well as an increase in the frequency of spontaneous excitatory postsynaptic currents and in NMDA receptor-mediated currents in lamina II neurons (Zhang et al., 2011). Similar effects are also described for IL1 β (Gustafson-Vickers et al., 2008). In this context, the preventive administration of both doses of MR309 (16 and 32 mg/kg) resulted in a complete abrogation of TNFα and IL1β expression upregulation in the spinal cord at 14 dpi, although only the high dose (32 mg/kg) was able to prevent the expression of both pro-inflammatory cytokines at 28 dpi. Our findings are in accordance with previous results showing a downregulation of spinal pro-inflammatory cytokines in the σ1R knockout SCI-mice compared to the wild-type mice after SCI (Castany et al., 2018), and overall, these results suggest that MR309 may modulate changes in the long term that may contribute to the attenuation of CNP development.

Our results with TNF-α and IL-1β are in accordance to those described for IL-6, another pro-inflammatory cytokine. IL-6 expression increases after spinal cord contusion (Nesic et al., 2002; Guptarak et al., 2013) and only those animals that develop central neuropathic pain are those showing an increase in IL-6 levels and the neutralization of the IL-6 receptor (IL6R) by the administration of specific antibodies has analgesic effects (Guptarak et al., 2013) Human primary monocyte-derived dendritic cells treated with LPS and ligands of σ1R cause inhibition of the production of IL-6, suggesting that IL-6 production is also under the σ1R regulation (Szabo et al., 2014), and it may be therefore modulated by MR309 as it is done with TNF-α and IL-1β after SCI.

Nowadays, the first line drugs of reference used to deal with neuropathic pain after spinal cord injury are the gabapentinoids gabapentin and pregabalin (Levendoglu et al., 2004; Siddall et al., 2006; Dworkin et al., 2007; Guy et al., 2014; Mehta et al., 2014). However, while several randomized controlled trials with patients suffering SCI-induced neuropathic pain have reported gabapentin and pregabalin efficacy in relieving main CNP symptoms (Levendoglu et al., 2004; Siddall et al., 2006; Cardenas et al., 2013), other clinical studies showed clear limitations. For instance, only those patients able to tolerate short- and long-term side effects had a significant pain relief (Putzke et al., 2002) and also, gabapentin lose efficacy in patients reporting neuropathic pain symptoms for more than 6 months (Ahn et al., 2003). In parallel with clinical evidences, gabapentin and pregabalin sometimes fail to provide analgesia in animal models (M’Dahoma et al., 2014; Kimura et al., 2016). Thus, the results obtained regarding gabapentinoid efficacy are controversial, and the use of σ1R antagonist may be an emerging suitable alternative to attenuate CNP development. Actually, experimental studies comparing σ1R antagonists with gabapentinoids show that while E-52862/MR309 inhibits mechanical allodynia after chronic constriction injury of the infraorbital nerve or mechanical hyperalgesia in the streptozotocin-induced neuropathic pain model, pregabalin treatment is ineffective (Gris et al., 2016). Likewise, the treatment with σ1R antagonists BD1047 and BD1063 in experimental models of neuropathic pain resulted in similar effects on mechanical allodynia alleviation (Entrena et al., 2009a,b; Son and Kwon, 2010) and even higher than gabapentin (Espinosa-Juárez et al., 2017). These results suggest that several σ1R antagonists (e.g., BD1047, BD1063, and E-52862/MR309) have analgesic effects which are similar or superior to gabapentinoids.

**Figure 5:**
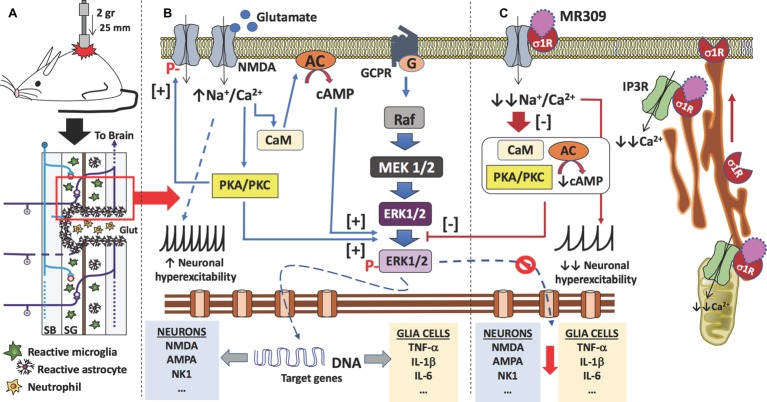
Proposed mechanism of action of MR309 to decrease the expression of inflammatory cytokines and the hyperexcitability of spinal neurons after contusion of the spinal cord. **(A)** Contusion of the spinal cord of the mouse causes a spinal cord injury which results in glutamate (Glut) release by necrotic death of neurons and glial cells, medullary parenchyma destructuring, progressive reactivation of microglia and astrocytes, and the formation of the glial scar that surrounds the area of injury. In this injured area, there is also an infiltration of blood-forming elements, such as neutrophils, lymphocytes, and monocytes, is evidenced. In the neurons and glial cells surviving in the spinal cord injury (red box), the excess of glutamate in the medullary parenchyma causes a set of molecular changes. **(B)** The glutamate can interact with the NMDA receptors present in neurons and glial cells, causing an increase in the influx of sodium and calcium ions. In both cell types, the increase in intracellular calcium ions causes the activation of calmodulin (CaM), so that the calcium-calmodulin complex in turn activates adenylate-cyclase (AC) with an increase in the synthesis of cAMP, the second messenger that causes the activation (phosphorylation) of ERK1/2. Also, calcium ions activate protein kinases (PKA and PKC), which in turn also facilitate the phosphorylation of ERK1/2. Phosphorylated ERK1/2 translocates to the nucleus and transcription of target genes begins. In the case of spinal nociceptive neurons, genes encoding neurotransmitter receptors (e.g., AMPA, NMDA, and NK1) as well as voltage-gated ion channels are transcribed. While in the case of glial cells (astrocytes and microglia), genes encoding pro-inflammatory cytokines are transcribed (e.g., TNF-α, IL-1β, and IL-6). On the other hand, the influx of sodium ions causes an increase in the neuronal excitability of the spinal nociceptive neurons, with an increase in action potentials by the spinothalamic tract toward the brain. Gene transcription of ion channels and receptors for neurotransmitters also facilitates neuronal hyperexcitability. Finally, activated protein kinases also cause phosphorylation of NMDA-receptors that will further contribute to central sensitization. **(C)** σ₁ receptor classified as a ligand-regulated molecular chaperone is activated under stress or pathological conditions and interacts with several neurotransmitter receptors and ion channels to modulate their function and its activity can be regulated by endogenous and/or synthetic compounds in an agonist-antagonist manner (Díaz et al., 2009). Upon σ₁R activation under stress or pathological conditions (SCI in this case), σ1R in the ER binds to IP3R to enhance Ca^2+^ influx into mitochondria and efflux into the cytosol. There is also redistribution of σ1R from mitochondria-associated endoplasmic reticulum membrane to peripheral endoplasmic membranes to bind ion channels, receptors (NMDA) or protein kinases which, in turn will produce a further increase in intracellular Ca^2+^. Therefore, σ1R antagonism by MR309 would result in a reduced Ca^2+^ cytosolic mobilization from ER stores (*via* PLC and IP3R) and in a reduced extracellular entry through NMDAR, favoring the access of negative regulators of the receptor such as Ca^2+^-CaM, finally resulting in an inhibition of Ca^2+^ dependent intracellular effectors such as PKC/PKA and CaMKII. This downstream modulation would modulate the ERK pathway by preventing the phosphorylation of ERK1/2, and consequently, the levels of gene expression of NMDAR and proinflammatory cytokines, together with a decrease in the overactivity of NMDAR (mediated by phosphorylations at S1303 and Y1472). All of these changes would decrease neuronal and glial hyperexcitability and thus produce an attenuation in central neuropathic pain development.

In summary, the present study has described spinal cord changes on three central sensitization-related mechanistic correlates, including extracellular mediators (i.e., TNF-α and IL-1β), membrane receptors/channels (i.e., NMDA receptors), and intracellular signaling cascades (i.e., ERK/pERK), and how these changes are prevented by MR-309 treatment. It is worth to note that these sustained antinociceptive effects of MR309 treatment on SCI-induced CNP development cannot be explained by a drug accumulating effect due to the pharmacokinetic profile of MR309 in rodents. MR309 maximum plasma concentration is achieved shortly after its administration to rodents (*tmax* = 15 min after i.p. administration to mice and rats), and it is quickly metabolized, having a short half-life (*t*1/2 = 1.4 h after administration to mice and rats). In fact, undetectable plasma levels were found by 6 h after its administration, its metabolites are inactive, and it does not accumulate in tissues, either in brain or in spinal cord (Romero et al., 2012; Vidal-Torres et al., 2014). In a parallel study, MR-309 at a single dose of 22.5 mg/kg i.p. showed levels of 2.8 μg/ml (plasma), 9.6 μg/g (brain), and 11.5 μg/g (spinal cord) 30 min after compound administration. The corresponding levels after repeated (12 days) administration were 2.6 μg/ml (plasma), 7.1 μg/g (brain), and 7.4 μg/g (spinal cord). Thus, the reduction in hypersensitivity of spinal cord-injured mice after MR309 treatment completion and throughout the experimental period could be explained by a “disease modification” effect associated with plastic changes (e.g., central sensitization) attenuation following a central injury. Hence, according to the results obtained in the present work and considering the physiopathology after spinal cord contusion (Yu and Yezierski, 2005; Wei et al., 2006; Latremoliere and Woolf, 2009; Vranken, 2013; Boadas-Vaello et al., 2016) (Figure 5) and the role of σ1R in the central pathological pain development (Castany et al., 2018), we propose that the pharmacological mechanism of repeated MR309 administration would be as follows (Figure 5). σ1R antagonist MR309 would bind to σ1Rs of both glial cells and nociceptive dorsal horn neurons, modulating NMDA receptors activity and blocking the activation of ERK and protein kinases. Consequently, the expression of pro-inflammatory cytokines (TNF-α and IL-1β) in glial cells would decrease, and also the upregulation of glutamate receptors as well as the phosphorylation of NMDA receptors in dorsal horn neurons would be prevented. Altogether, these effects would be translated into a significant reduction in neuronal hyperexcitation, and therefore preventing central neuropathic pain development after spinal cord injury (Figure 5). Finally, it is worthy to note that although the expression of molecules inducing neuropathic pain in the lumbar region has not been evaluated, similar results to those observed in the area of primary injury are expected. It has been shown a remote reactivation of microglia cells in the lumbar regions after a thoracic spinal cord injury and the reactive microglia cells of these lumbar regions secrete pro-inflammatory cytokines (e.g., TNF-α, IL-1β, and IL-6). The production of these mediators in remote regions from SCI may be associated with the development of below-level neuropathic pain (Detloff et al., 2008), which would be also modulated by repeated MR309 treatment. Here we have observed that repeated treatment with MR309 after thoracic contusion causes a decrease in the expression of inflammatory cytokines *via* decreased phosphorylation of ERK1/2 in the injury-site, but it may not be ruled out that this treatment would also exert a decrease of remote microglia reactivity and subsequent inflammatory cytokines release in lumbar regions.

## Conclusion

In conclusion, our findings indicate that the preventive treatment by repeated administration of the σ1R antagonist (MR309) after injury attenuates CNP development in wild-type CD-1 Swiss female mice during the acute phase of SCI. MR309 pharmacological effects are associated with the prevention of upregulation of central sensitization-related biomarkers (pERK, pNR2Bs, IL1-β, and TNF-α). On the basis of these results, repeated MR309 treatment following SCI is suggested to be a potential pharmacological strategy to prevent pathological pain development after spinal cord injury.

## Data Availability

All datasets generated or analysed for this study are included in the manuscript and/or the Supplementary Files.

## Author Contributions

All authors listed above have contributed sufficiently to be included as authors. PB-V, EV, XC, DZ, and MM conceived the experiments, contributed to the analysis and/or interpretation of data, the critical discussion of the results and the elaboration of the manuscript, and obtained funding for the study. SC has directly participated in the execution of the experimental work, both behavioral and biochemical assays, and has contributed to the critical discussion of the results and elaboration of the manuscript. All the authors have revised the work critically for important intellectual content and approved the final version to be published. Also, agreed to be accountable for all aspects of the work in ensuring that questions related to the accuracy or integrity of any part of the work are appropriately investigated and resolved.

### Conflict of Interest Statement

XC, DZ, and MM are full-time employees of ESTEVE. The authors have no other relevant affiliation or financial involvement, have received no payment in preparation of this manuscript or have any conflict with the subject matter or materials discussed in the manuscript apart from those disclosed.

The remaining authors declare that the research was conducted in the absence of any commercial or financial relationships that could be construed as a potential conflict of interest.
