# Supplementary material for: Repeated Sigma-1 Receptor Antagonist MR309 Administration Modulates Central Neuropathic Pain Development After Spinal Cord Injury in Mice
Source: Front Pharmacol. 2019 Mar 22;10:222. doi: 10.3389/fphar.2019.00222 (PMC6439356; doi:10.3389/fphar.2019.00222)
Supplement: Supplementary file 1 [file Data_Sheet_1.pdf]

Supplementary Material

Repeated Sigma-1 receptor antagonist MR309 administration modulates central neuropathic pain development after spinal cord injury in mice

Sílvia Castany[a,b], Xavier Codony[b], Daniel Zamanillo[b], Manuel Merlos[b], Enrique Verdú [a,1], and Pere Boadas-Vaello [a,1,*]

[a] Research Group of Clinical Anatomy, Embryology and Neuroscience (NEOMA), Department of Medical Sciences, Universitat de Girona (UdG), Girona, Spain.

[b] Esteve Pharmaceuticals, Drug Discovery and Preclinical Development, Parc Científic de Barcelona, Barcelona, Spain.

[1] Authors Pere Boadas-Vaello and Enrique Verdú Navarro contributed equally to this work

*** Correspondence:** Pere Boadas Vaello : pere.boadas@udg.edu

Supplementary Figures

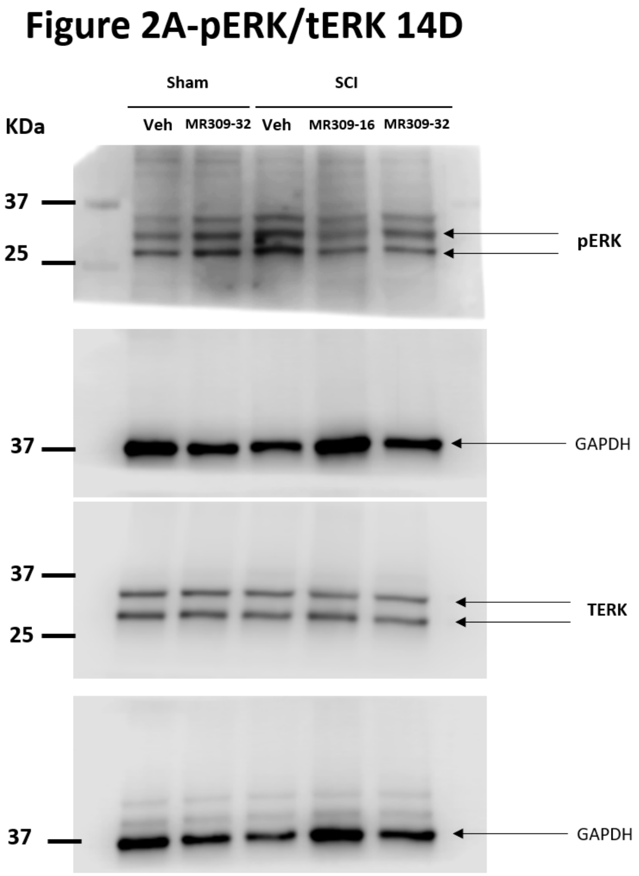

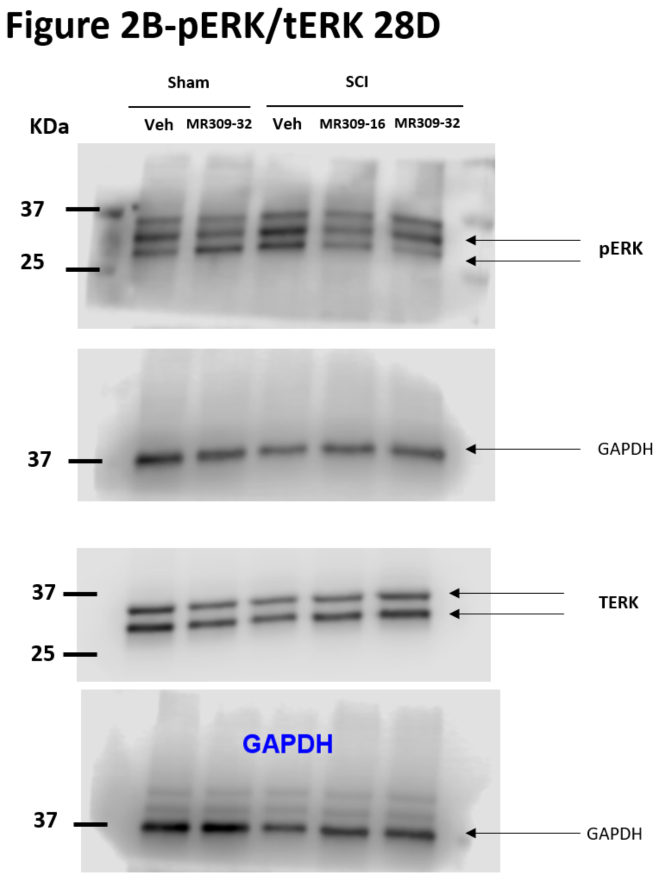

Supplementary Figure S1. Original scanned full blots for pERK, tERK and GAPDH shown in Figure 2 A,B.

Figure 3A-Y1472 NR2B 14D

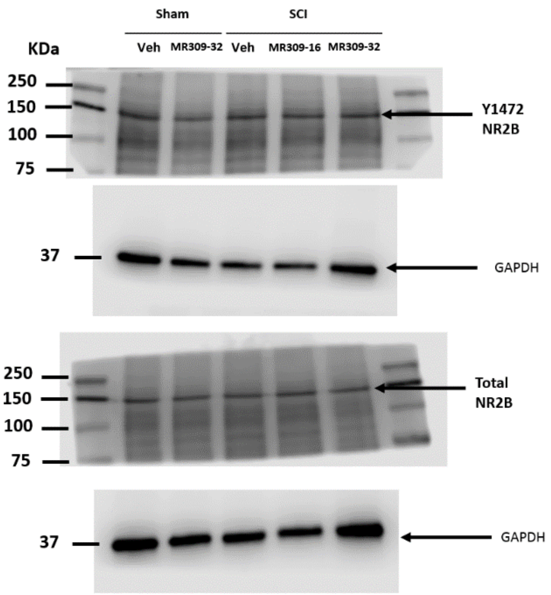

Figure 3B-Y1472 NR2B 28D

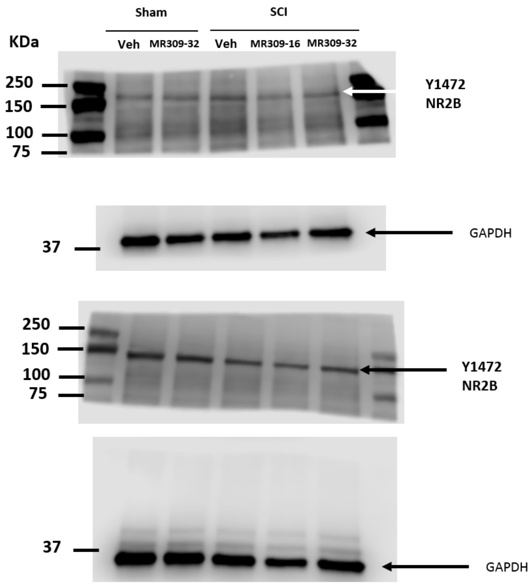

Figure 3C-S1303 NR2B 14D

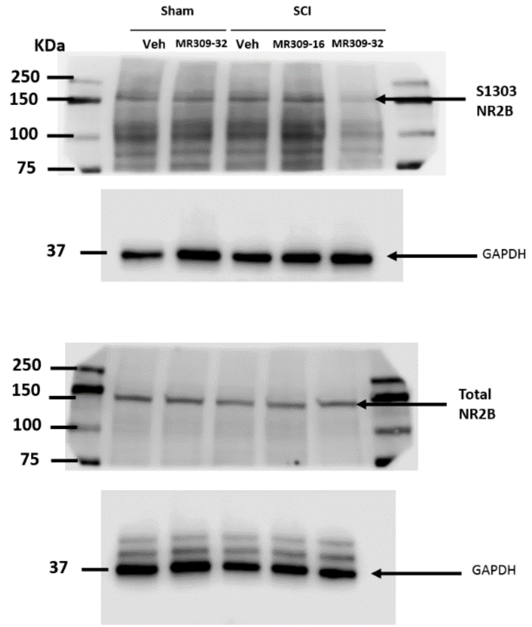

Figure 3D-S1303 NR2B 28D

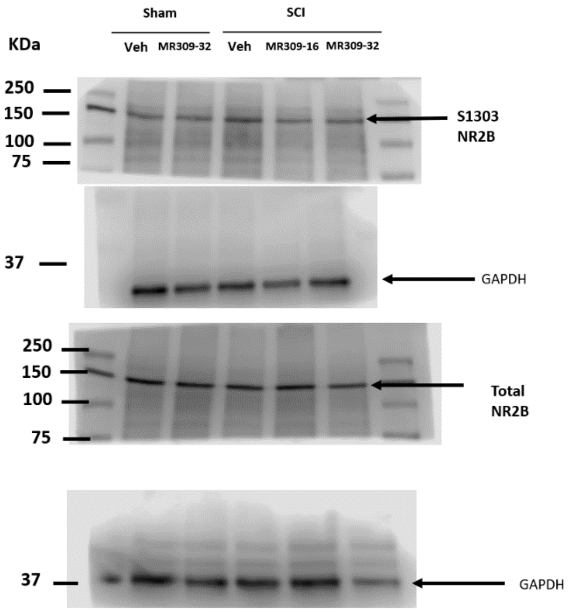

Supplementary Figure S2. Original scanned full blots for Y1472 and S1303 phosphorylation of NR2B, NR2B total and GAPDH shown in Figure 3 A,B,C,D.

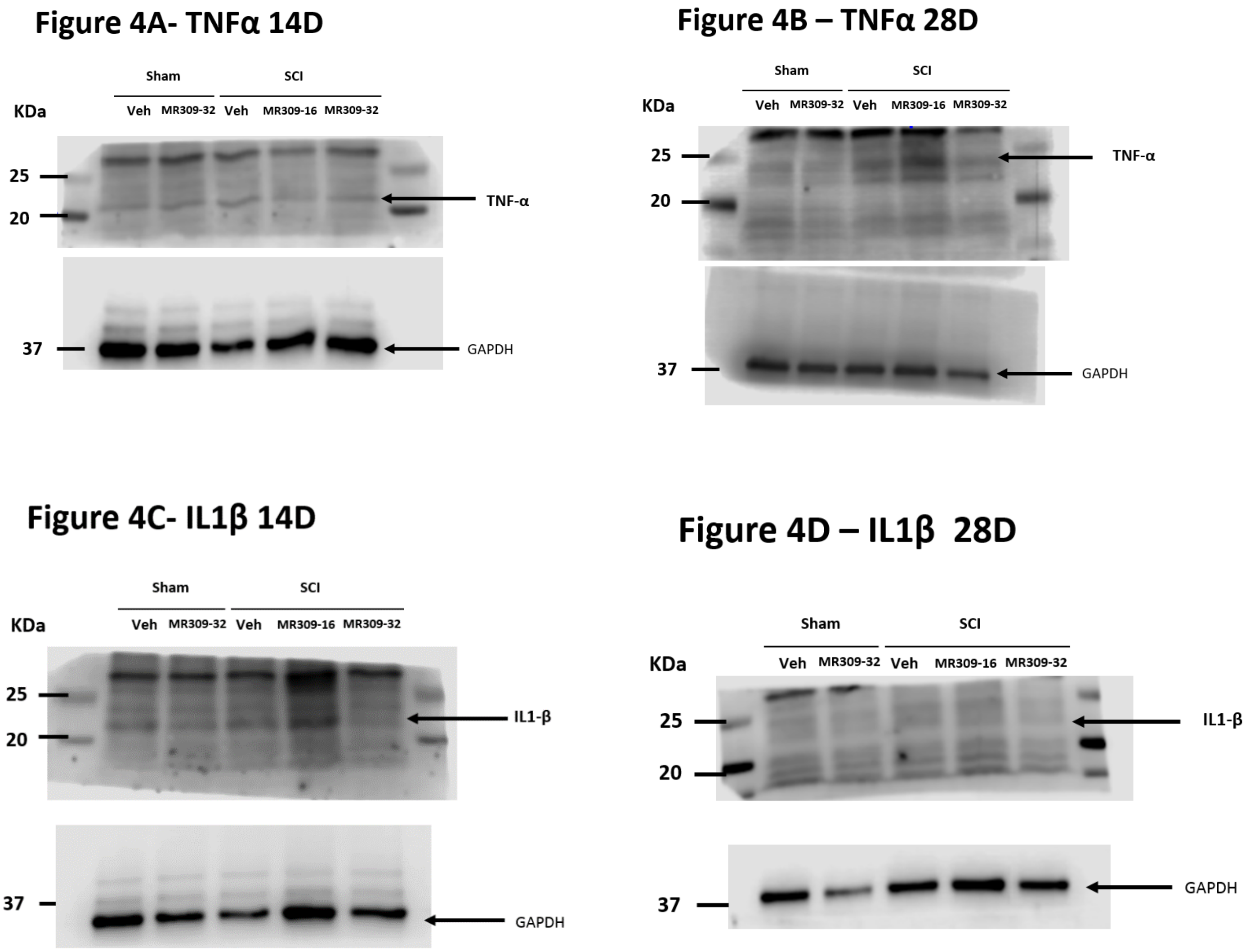

Supplementary Figure S3. Original scanned full blots for TNF-α, IL1β and GAPDH shown in Figure 4 A,B,C,D.
